# The research life cycle and the health sciences librarian: responding to change in scholarly communication

**DOI:** 10.5195/jmla.2017.110

**Published:** 2017-01

**Authors:** Andrea M. Ketchum

The Internet and digital technologies have profoundly affected scholarly communication, publishing, collaborative research, literature searches, and management of digital assets and data [1, 2]. In turn, our views of the research life cycle have changed. What does this mean for librarians in the health sciences who support or even actively participate in clinical research?

## EVOLUTION OF THE SCHOLARLY COMMUNICATION LIFE CYCLE

In 2002, the United Nations International Scientific Information System (UNISIST) model of scientific and technical communication at last recognized the effect of the Internet and digital technologies on the exchange of scientific ideas (described by Söndergaard et al. [1]). In the updated UNISIST life cycle for scholarly communication, primary communication flows from knowledge producers to knowledge users. Additions in the updated version follow the three primary research sources listed below. While the primary sources might not have changed, the additions introduced electronic communications [3]:

1. Informal communication (written or oral), such as letters and lectures: additions included Internet, online public access catalogs (OPACs), search engines, email, newsgroups, email discussion lists, preprints, webinars, and e-conferencing
2. Formal communication (published and unpublished documents): additions included bibliographic databases, e-journals, and preprints
3. Tabulations of quantitative data not presented as flowing text: additions included online collaborative spreadsheets and data manipulation and analysis tools

It is interesting that the UNISIST model is built upon the research products themselves, not the research activities. By 2002, digital tools and electronic access to what were previously print or in-person resources had expanded the range of materials available with almost instant delivery, all of which increased partnering opportunities for librarians and researchers.

In contrast to UNISIST, Björk used business process modeling in 2005 to analyze variables such as cycle time, quality, or cost. Björk created a hierarchical list of stages for the entire research publication process, with possible inputs or activities from represented stakeholders [2]. Björk asserted that this might have been the first time formal modeling was used to comprehensively map the research publishing system in the digital culture. Björk’s 2007 extensive model of the research life cycle was built upon four primary activities: (1) performing research, (2) communicating knowledge, (3) applying knowledge, and (4) evaluating research or the researcher. Each primary activity included its own set of activities, incorporating both traditional and digital tools to a greater extent over time. The activity “communicating knowledge” was especially affected by the Internet with the inclusion of email in the category “personal communication” and online publishing in the category “professional dissemination of scientific results.” Ultimately, Björk’s scholarly communication life cycle was depicted in a forty-eight-page document with thirty-three diagrams in hierarchies up to seven levels deep [4].

## DIGITIZATION OF THE RESEARCH WORK-FLOW AND LIBRARIAN EXPERTISE

One interesting outcome of modeling the research life cycle is a growing recognition that digitization of the research work-flow expands collaborative opportunities for librarians, from developing effective literature searches to improving research impact. To add value during the research life cycle, here is a brief list of topics and related questions that researchers might ask and that librarians should be prepared to answer:

1. Funding
  - How can I comply with public access policies?
  - How can the following tools help me manage my funded research?
    - ORCID, a unique author identifier [5]
    - My NCBI, an online system for managing grant documentation required by the National Institutes of Health (NIH) [6]
    - SciENcv, an online biosketch management tool in My NCBI for researchers associated with federal grants [7]
    - My Bibliography, the publications tool linked to My NCBI [8]
2. Literature search
  - Which databases are most relevant for my research topic?
  - What are the best search terms to use across databases?
  - When can I rely on Google Scholar, and why do I need a librarian’s help to search subject-specific bibliographic databases? What are controlled vocabularies?
3. Open access publishing
  - What is the difference between open access and public access?
  - How can I find a good open access journal? How do I pay the article processing charges?
  - How can I recognize predatory publishers?
4. Data management
  - How should I name my data files? Why should I standardize my approach?
  - What repository should I use to deposit data that I generate? How do I prepare my data for submission? How do I get credit for sharing my data?
5. Copyright
  - How can I retain my copyrights, and does that affect my choice of publishers?
  - Is there any advantage to surrendering my copyrights to a traditional publisher?
6. Documentation
  - How can I ensure that my study is replicable?
  - Should I use an electronic notebook?
  - How do I document my datasets?
  - When should I acknowledge funding sources and conflicts of interest?
7. Dissemination
  - In which journal should I publish?
  - What if my paper is rejected? How do I know which journal to try next?
  - How can I promote my published article? What type of promotion reaches more readers?
8. Impact metrics
  - What do I need to know about impact metrics?
    What is the difference between traditional bibliometrics and newer altmetrics?
  - How useful are altmetrics?
  - What mix of metrics should I use to demonstrate my productivity as a researcher?

## STRATEGIC PLANNING AND THE RESEARCH LIFE CYCLE

With so many areas within the librarian’s scope of expertise, it is not surprising that some medical libraries employ research life cycle analysis for strategic planning. Using a localized model, librarians can map resources and services to research tasks or needs specific to their institutions, highlighting the capabilities of their librarians to better attract and serve researchers. Marketing through liaison librarians can increase collaboration with researchers and their departments, as well as make more efficient and effective use of researcher time—a benefit for the entire institution.

For example, science and health sciences librarians at the University of North Carolina–Chapel Hill (UNC-CH) identified new library services or roles for librarians in the research setting [9]. Their analysis revealed five phases of the life cycle to which library services were then mapped:

Idea Development → Funding → Proposal → Conducting → Disseminating

Concept maps created by nine science librarians resulted in eighty-four services mapped to each of the five phases of the life cycle. Potential services included identifying funding opportunities, helping with digitization projects, and helping with scholarly communication tasks, such as assisting researchers in depositing to an institutional repository. Faculty members and graduate students favored four services: providing grant information, finding background literature, navigating repository options, and helping with the NIH public access policy. Services perceived as popular may vary by institution or location and deserve additional study to gauge the effectiveness of the institution-specific model.

In contrast to the UNC-CH localized model of the research life cycle, Kramer and Bosman identified six main phases, based on a global survey of researchers’ use of digital tools [10]:

Discovery → Analysis → Writing → Publication → Outreach → Assessment

Groups of digital tools define each phase, which is analogous to Björk’s model. Of note, in their “101 Innovations in Scholarly Communication” circular chart [10], all activities are illustrated with purely digital or computer-based applications without reference to the original human activity.

## DATA MANAGEMENT AND PUBLIC ACCESS POLICIES

The current emphasis on data management stems from recent federal public access policies. Furthermore, the data life cycle is now viewed as complementary to the research life cycle. Tenopir et al. used a simple model to show relationships between the data and research life cycles [11].

Information management is at the heart of librarian expertise, often in the form of knowledge of metadata for cataloging, so it should not be surprising that librarians have been involved in major data repository projects such as Dryad, where researchers can deposit data and receive a data-specific digital object identifier (DOI) to submit to the publisher alongside the manuscript. The manuscript can then be deposited later, either in Dryad or a different repository of choice with its own DOI, assuring credit for each work product.

Librarians also create customizable data management plan (DMP) templates for use in grant applications. For example, the DMPTool is online software for automatically generating customized DMPs [12]. Librarians can collaborate with their institutions’ schools, departments, and centers to train researchers how to write a DMP and make it easy to complete research forms required by federal, state, or other agencies.

## AN EXPANDED VERSION OF THE RESEARCH LIFE CYCLE

Additional research life cycles include a version of a 1979 cycle updated for digital use at the University of Mexico–Valencia [13] and experimental work integrating repositories from Assante et al. in Italy [14]. Based on these studies and tabulation of the various components, an expanded version of the research life cycle includes seven phases:

Idea → Study Preparation → Research → Write → Publish → Disseminate → Assess

## THE CLINICAL RESEARCH LIFE CYCLE REQUIRE ADDITIONAL PHASES

The clinical research life cycle, however, is more complex. A model must incorporate additional components for clinical studies and account for longer time frames, especially for multifaceted clinical trials or multicenter studies. The Institute of Medicine’s 2015 report, *Sharing Clinical Trial Data: Maximizing Benefits, Minimizing Risk,* dedicated a chapter to identifying clinical trial research stages to support data-sharing recommendations [15], which provides a new layer of value to the framework for data management purposes:

Trial Design and Registration → Participant Enrollment → Study Completion → Publication → Regulatory

These phases easily fit into the generic research life cycle by extending each where necessary with appropriate details. In practice, the Pulmonary Translational Research Core at the University of Pittsburgh School of Medicine has devised a research life cycle with nine phases including three elements found in the basic model: idea development, publication, and dissemination [16]. Another four categories of the Pittsburgh clinical model are driven by regulatory and human subject requirements and would most likely fit in the early “Study Preparation” category: assess feasibility, prepare regulatory documents, submit and obtain institutional review board and regulatory approvals, and recruit subjects. The final two components belong in the “Research” category of the basic model: implement the study and close the study.

Thus, existing research life cycles can be modified to accommodate clinical research with careful thought at the local level. During strategic planning, appropriate tasks for librarians—such as literature searching, data management, grants management, and publishing—could be matched with librarian roles that have corresponding expertise.

## CONCLUSION

Librarians strengthen the research community and facilitate scholarly communication. They specialize in managing digital resources and teach researchers how to use tools that reduce their workload. As competition and expectations from funders, publishers, institutions, and the public increase, library services and training in research management must expand to meet the needs of the research community. By planning strategically, librarians in the health sciences can demonstrate their value to their institutions as knowledgeable information resources who are able to support all aspects of the contemporary and ever-evolving research life cycle.
